# Phloem-specific expression of a melon *Aux/IAA* in tomato plants alters auxin sensitivity and plant development

**DOI:** 10.3389/fpls.2013.00329

**Published:** 2013-08-23

**Authors:** Guy Golan, Rotem Betzer, Shmuel Wolf

**Affiliations:** The Robert H. Smith Faculty of Agriculture, Food and Environment, Otto Warburg Minerva Center for Agricultural Biotechnology, The Robert H. Smith Institute of Plant Sciences and Genetics in Agriculture, The Hebrew University of JerusalemRehovot, Israel

**Keywords:** *Aux/IAA*, auxin response, phloem, root development, *Solanum lycopersicum*

## Abstract

Phloem sap contains a large repertoire of macromolecules in addition to sugars, amino acids, growth substances and ions. The transcription profile of melon phloem sap contains over 1000 mRNA molecules, most of them associated with signal transduction, transcriptional control, and stress and defense responses. Heterografting experiments have established the long-distance trafficking of numerous mRNA molecules. Interestingly, several trafficking transcripts are involved in the auxin response, including two molecules coding for auxin/indole acetic acid (Aux/IAA). To further explore the biological role of the melon *Aux/IAA* transcript *CmF-308* in the vascular tissue, a cassette containing the coding sequence of this gene under a phloem-specific promoter was introduced into tomato plants. The number of lateral roots was significantly higher in transgenic plants expressing *CmF-308* under the AtSUC2 promoter than in controls. A similar effect on root development was obtained after transient expression of *CmF-308* in source leaves of *N. benthamiana* plants. An auxin-response assay showed that *CmF-308-transgenic* roots are more sensitive to auxin than control roots. In addition to the altered root development, phloem-specific expression of *CmF-308* resulted in shorter plants, a higher number of lateral shoots and delayed flowering, a phenotype resembling reduced apical dominance. In contrast to the root response, cotyledons of the transgenic plants were less sensitive to auxin than control cotyledons. The reduced auxin sensitivity in the shoot tissue was confirmed by lower relative expression of several *Aux/IAA* genes in leaves and an increase in the relative expression of a cytokinin-response regulator, *TRR8/9b*. The accumulated data suggest that expression of *Aux/IAA* in the phloem modifies auxin sensitivity in a tissue-specific manner, thereby altering plant development.

## Introduction

It is now evident that phloem sap contains a wide range of mRNA molecules (Vilaine et al., 2003; Omid et al., 2007; Buhtz et al., 2008; Zhang et al., 2009). Transcription profiling of the phloem sap of cucurbit plants has enabled the identification of thousands of mRNA molecules, as well as tRNAs, and small and microRNAs. Intuitively, one would think that all of these phloem-sap molecules are destined for long-distance movement. However, heterografting experiments indicate that only a small proportion of these molecules are capable of long-distance movement (Omid et al., 2007). Interestingly, numerous long-distance trafficking mRNA molecules have been characterized as coding for proteins involved in signal transduction mediated by gibberellin (Haywood et al., 2005), gibberellins and cytokinin (Banerjee et al., 2006) or auxin (Omid et al., 2007; Notaguchi et al., 2012).

We previously examined the ability of 43 melon phloem sap mRNA molecules to traffic long distances using melon–pumpkin heterografting experiments. Interestingly, only six of the examined melon transcripts were identified in the pumpkin scion (Omid et al., 2007). Annotation of these transcripts revealed that two of them were auxin/indole acetic acid (*Aux/IAA*) and one was *small auxin-up RNA* (*SAUR*), while the other three encoded “hypothetical proteins” (Omid et al., 2007).

*Aux/IAA* is a large family of early auxin response genes with 29 and 26 members in *Arabidopsis* and tomato, respectively (Overvoorde et al., 2005; Audran-Delalande et al., 2012). These genes encode transcriptional repressors of auxin response factor (ARF), thereby preventing transcription of genes controlled by these ARFs. Interaction of auxin with transport inhibitor response 1 (TIR1) and auxin F-box protein (AFB) forms part of the SCF ubiquitin-ligase (SCF^TIR1^) complex which catalyzes ubiquitin-mediated degradation of Aux/IAA (Teale et al., 2006). It has recently been shown that efficient auxin binding requires assembly of Aux/IAA and TIR1 proteins. The various combinations of TIR1–Aux/IAA complexes interact with auxin with a wide range of affinities (Calderon Villalobos et al., 2012).

The encoded Aux/IAA proteins are highly conserved in four distinct domains (Woodward and Bartel, 2005). Domain I is a transcriptional repressor, domain II is critical for Aux/IAA instability, and domains III and IV are involved in homodimerization and heterodimerization with other Aux/IAA proteins or with ARF (Reed, 2001). Genetic screens have identified *Arabidopsis* plants with mutations in various *Aux/IAA* genes that result in changed morphology. Most of these primary mutations were located in the highly conserved domain II, which is responsible for protein degradation. Such mutations result in stable proteins and gain-of-function phenotypes (e.g., insensitivity to auxin). An excellent example is the *solitary root* (*slr*) mutant which has reduced sensitivity to auxin (Fukaki et al., 2002, 2005; Vanneste et al., 2005). This dominant mutant completely lacks lateral roots and is also defective in root-hair formation and in gravitropic responses of its roots and hypocotyls. Map-based cloning and isolation of an intragenic suppressor mutant revealed that *SLR* encodes IAA14, a member of the Aux/IAA protein family (Fukaki et al., 2002).

Substantial phenotypic changes were also observed in transgenic tomato plants into which an antisense form of *SlIAA9*, another member of the Aux/IAA protein family, was inserted under the control of the *CaMV-35S* promoter (Wang et al., 2005). Significant reduction in the accumulation of *IAA9* transcript was associated with altered leaf morphology, increased number of lateral shoots, parthenocarpic fruit development, enhanced hypocotyl/stem elongation and increased leaf vascularization. Auxin dose-response assay of cotyledon explants confirmed that *SlIAA9* antisense plants are more sensitive to exogenous auxin than control plants (Wang et al., 2005). Interestingly, expression of *SlIAA3* was higher in *SlIAA9*-antisense than control plants. Consistent with these results, roots of tomato plants in which *SlIAA3* was silenced by expression of its antisense form were less sensitive to auxin than control roots (Chaabouni et al., 2009). Nevertheless, these antisense plants were also characterized by a higher number of lateral shoots.

The effect of *Aux/IAA* overexpression on plant development has been only scarcely examined. Transgenic *Arabidopsis* plants expressing the *Vitis vinifera IAA9* (Fujita et al., 2012) or *IAA19* (Kohno et al., 2012) grew somewhat faster but were similar to control plants in terms of morphological characteristics. On the other hand, overexpression of *AtIAA20*, *AtIAA30* or *AtIAA31* in transgenic *Arabidopsis* plants caused auxin-related aberrant phenotypes including semi-dwarfism, malformed vasculature and inhibition of primary root growth (Sato and Yamamoto, 2008). Significant inhibition of primary root length and increased number of adventitious roots were also observed when *AtIAA17* was overexpressed in transgenic *Arabidopsis* plants (Worley et al., 2000).

It is important to note that all of these studies included transgenic plants expressing the *Aux/IAA* gene under the control of the *CaMV-35S* promoter. To better understand the biological function of *Aux/IAA* in the phloem, *CmF-308—*the long-distance-trafficking melon *Aux/IAA*—was expressed in the phloem of tomato plants under control of the *AtSUC2* promoter. The phenotype of these plants indicated a modified auxin response while assays established tissue-specific alterations in auxin sensitivity. It is therefore concluded that the *Aux/IAA* gene product can exert its influence over plant developmental processes while being expressed in the phloem.

## Materials and methods

### Plant material

Tomato (*Solanum lycopersicum*), melon (*Cucumis melo*) and *Nicotiana benthamiana* plants were grown in a temperature-controlled greenhouse at 25–28/18–20°C (day/night, respectively), under natural sunlight. For hydroponic experiments, *N*. *benthamiana* was grown in trays containing coconut mixture. Two-week-old seedlings were transferred to containers (390 × 330 × 163 mm) with a nutrient solution containing 6 mM KNO_3_, 4 mM Ca(NO_3_)_2_, 2 mM KH_2_PO_4_, 0.03 mM EDFS [ethylenediamine tetraacetic acid iron (III) sodium salt], 0.5 μM CuSO_4_, 0.5 μM H_2_MoO_4_, 2 μM MnSO_4_, 50 μM KCl, 2 μM ZnSO_4_. The seedlings were transplanted into 5-cm plastic tubes that were fitted into holes drilled into the cover of the container such that the roots were inside the solution and the shoots above the cover. The nutrient solution was replaced twice weekly and was continuously aerated with an aquarium pump.

Transgenic tomato plants containing the *pAtSUC2:GFP* insert were employed to verify promoter activity. GFP fluorescent was visualized using confocal microscopy (CLSM510, Zeiss, Jena GmBH).

### RNA isolation and RT-PCR

Total RNA was extracted from different tissues of tomato and melon plants using Tri-reagent (Sigma, http://www.sigmaaldrich.com/) according to the manufacturer's protocol. cDNA was prepared from the same amounts of RNA (1 μg) per sample pretreated with 1 unit μg^−1^ of RQ1 DNAse (Promega, http://www.promega.com), using the Verso cDNA synthesis kit (Thermo Scientific, http://www.thermoscientific.com). A 2-μl aliquot of cDNA was taken for PCR amplification. Real-time RT-PCR was carried out using 0.5 μl of 2.5 pmol of each primer (Table A1), 4 μl cDNA and 5 μl ABsolute™ Blue QPCR SYBR® Green ROX Mix. PCR conditions were as follows: 95°C for 10 s, 59°C for 15 s and 70°C for 25 s, repeated 45 times. The obtained cycle temperature (CT) values were analyzed with Rotor-Gene 6000 Series software by averaging the three independently calculated normalized expression values of the triplicates. The calculated numerical values were divided by the values obtained for the housekeeping gene *tubulin* in each respective sample.

### Laser-capture microdissection

Expression of *CmF-308* in specific cells of melon plants was determined using a laser-capture microdissection system according to Gil et al. (2011). In short, trimmed leaf discs were fixed in Farmer's fixative (3:1 v/v ethanol:acetic acid). Fixed tissue was dehydrated in a graded series of ethanols, after which it was incubated in isopropyl alcohol inside a microwave histoprocessor. Wax impregnation was performed under vacuum.

Cross sections (12 μm) were cut on a rotary microtome (Leica RM2245), floated in water at 42°C to stretch ribbons and incubated on membrane microscope slides. Prior to laser microdissection, slides were deparaffinized twice for 10 min each in 100% Histoclear (Gadot), followed by one wash in 100% ethanol (2 min) and then air-drying in the hood for 5 min. For microdissection, a PALM Laser Microbeam Instrument (Zeiss) was employed. Specific mesophyll and vascular bundle cells were isolated separately by the laser microbeam and collected into the lid of a 0.5-mL reaction tube (Zeiss) filled with 30 μ L ethanol, and placed in a holder located just above the slides.

RNA was extracted and isolated from each reaction tube using the PicoPure RNA isolation kit according to the manufacturer's protocol (Arcturus, http://www.moleculardevices.com/). Isolated samples were treated with the RNase-free DNase set kit (Qiagene, http://www.qiagen.com/). RNA was amplified twice and reverse-transcribed using MessageBOOSTER™ whole transcriptome cDNA synthesis kit (Epicentre, http://www.EpiBio.com/).

### Cloning and plant transformation

The coding sequence plus 18bp of the 5' untranslated region (UTR), without the stop codon and the 3' UTR, of the *CmF-308* mRNA was amplified from melon (*Cucumis melo*) cDNA by PCR and then cloned into the pTZ57R vector (Fermentas, 2886 bp) using the T-A ligation protocol. The gene was further restricted by Xho1 and Kpn1 and cloned into the pART7 vector upstream of three HA-tag repeats. The fused *CmF-308-3xHA* fragment was amplified by PCR, restricted with Sma1 and Hind3, and then cloned into the pART27 binary vector (Gleave, 1992) downstream of the *AtSuc2* promoter (Truernit and Sauer, 1995) and upstream of the *Ocs* terminator. Cotyledon transformation was performed according to McCormick (1991).

### Agro-infiltration

*Agrobacterium tumefaciens* (strain GV3101) containing the pART27 vector was grown overnight at 28°C in Luria-Bertani medium containing 50 mg L^−1^ gentamycin and spectinomycin. The culture was precipitated by centrifugation for 10 min at 3000 g and then resuspended in inoculation buffer containing 50 mM MES, 0.5% (w/v) glucose, 2 mM Na_3_PO_4_, and 200 μM acetosyringone to an OD600 of 0.5. The bacteria were then infiltrated into *N. benthamiana* leaves using a syringe without a needle. Shoots and roots of the infiltrated plants were collected 14 days after infiltration and dry weight was determined after 4 days at 65°C.

### Auxin dose-response assay

Auxin-response assay was performed according to Wang et al. (2005). Tomato seeds were sown in sterile ½ MS medium containing 2.2 g L^−1^ MS and 30 g L^−1^ sucrose. Cotyledons (9 days old) were dissected and placed on MS medium containing 4.4 g L^−1^ MS and 30 g L^−1^ sucrose with varying concentrations of NAA. Root development from the cut cotyledons was determined 12 days after dissection.

The root-development response to auxin was analyzed in developing seedlings. Seeds were sown on germination papers placed in plastic bags supplemented with tap water. Germinated seeds were transferred to new germination papers soaked in solutions with varying concentrations of NAA, in plastic bags. The bags were kept in the dark at a temperature of 25 ± 2/18 ± 2°C for 10 days, after which the length of the primary root was measured.

## Results

### *CmF-308* transcript accumulates in the vasculature of melon plants

**Figure 1:**
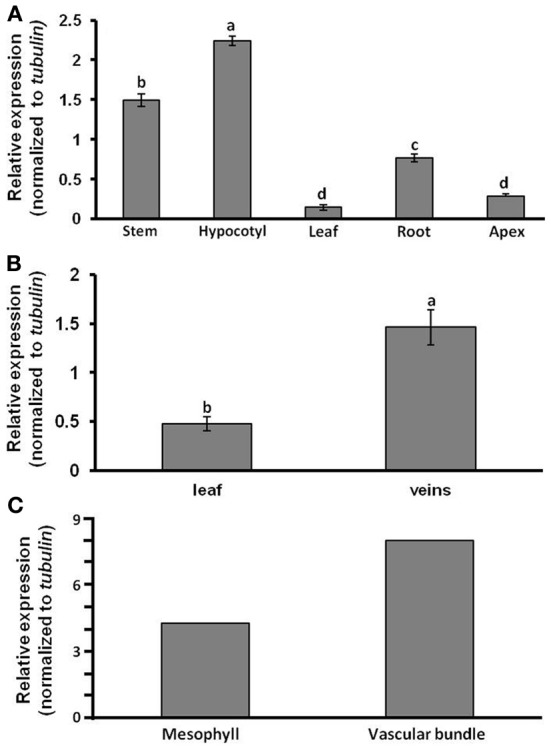
**Relative expression levels of *CmF-308*, as determined by real-time RT-PCR.** Relative expression was compared in various melon plant organs **(A)**, in veins and leaf lamina **(B)** and in mesophyll and vascular bundle cells isolated by laser-capture microdissection system **(C)**. Leaf discs and single cells were collected from the youngest mature source leaf (no. 4). Data represent means of three to six replications (±SE). Different letters indicate significant differences between plant organs or cell types at *p* < 0.05 by Tukey's HSD-test.

Following our finding that the melon phloem-sap transcript *CmF-308* is capable of long-distance trafficking, our initial set of experiments was aimed at identifying its level of accumulation in various tissues of melon plants. Quantitative (q) RT-PCR analyses revealed highest relative expression of this transcript in the hypocotyls and stems of young melon seedlings, with significantly lower relative expression in roots, leaves and shoot apices (Figure 1A). To further verify that *CmF-308* accumulates in tissues enriched with vascular bundles, veins were separated from young mature leaves. Here again, relative expression of *CmF-308* in the veins was three times higher than that in the rest of the leaf (Figure 1B). Predominant accumulation of *CmF-308* in vascular cells was confirmed by collection of specific cell types using the laser-capture microdissection system (Figure 1C).

### Transient expression of *CmF-308* modifies root development in *N. benthamiana* plants

**Figure 2:**
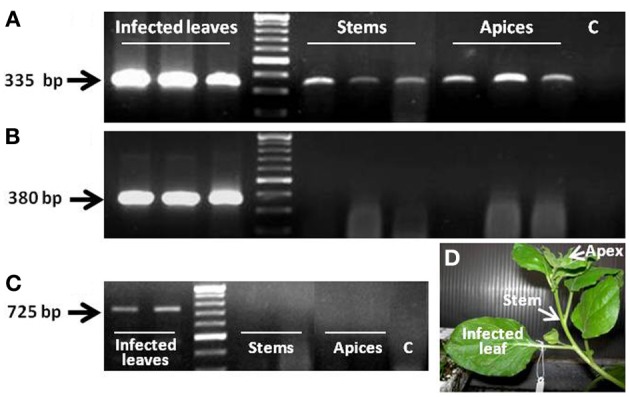
**Presence of *CmF-308* transcript in the stems and apices of *N. benthamiana* plants 48 h after agroinfiltration of source leaves with plasmid containing the coding sequence of this transcript, indicating long-distance movement of the transcript (A).** Absence of the *gfp* transcript from the apices and stems and presence of the transcript in the agroinfiltrated leaf **(B)**. **(C)** Presence of the agrobacterium in the infected leaf 48 h after agroinfiltration. C, negative control RT-PCR with no cDNA template. **(D)** Picture of the infiltrated *N. benthamiana* plant.

As indicated, *F-308* codes for Aux/IAA, one of the auxin-response regulators. The next set of experiments was aimed at studying the potential functioning of this gene product in the phloem, as a component of the auxin response pathway. A cassette harboring the coding sequence of *CmF-308* under the *AtSUC2* promoter was agroinfiltrated into leaves of *N. benthamiana* plants (Figure 2D). Presence of the melon transcript in the apices of these *N. benthamiana* plants 48 h post-infiltration established that this transcript is indeed capable of long-distance movement (Figure 2A). It is important to note that the *gfp* transcript that served as a control was absent from the stem and shoot apices, indicating that long-distance movement of RNA molecules is not a general phenomenon (Figure 2B). PCR analysis failed to detect segments of the binary plasmid, inserted into the *Agrobacterium*, outside the infiltrated leaf, indicating that the bacteria were restricted to this tissue during the first 48 h post-infiltration (Figure 2C).

**Figure 3:**
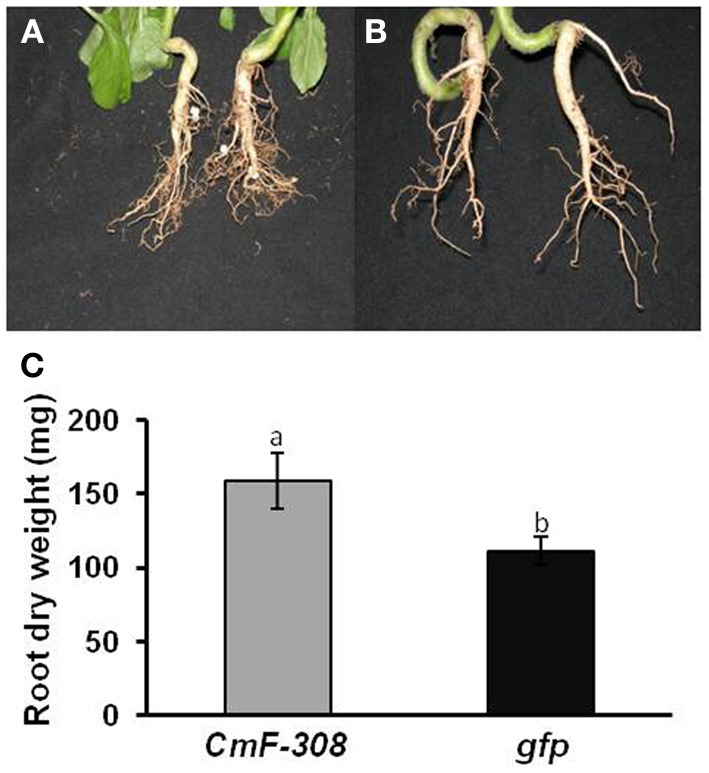
**Effect of transient expression of *CmF-308* on root mass of *N. benthamiana* plants.** Roots of pot-grown plants agroinfiltrated with plamids containing the coding sequence of *CmF-308* **(A)** or *gfp* **(B)**. Pictures were taken 14 days after infiltration of the respective plasmids into the source leaves. **(C)** Root mass of hydroponically grown *N. benthamiana* plants 14 days after infiltration of the *CmF-308* or *gfp* coding sequence into the source leaves. Data represent means of eight replications ± SE. Different letters indicate significant differences between plant lines at *p* < 0.05 by Student's *t*-test.

Interestingly, root mass of plants infected with the plasmid containing *CmF-308* was significantly higher than that of plants into which the control *CaMV35S:GFP* cassette was inserted (Figure 3). The enhanced root growth in plants transiently expressing the *CmF-308* gene product was evident both in pot-grown (Figures 3A vs. 3B) and hydroponically grown (Figure 3C) plants. The higher root weight of plants expressing the *CmF-308* gene product was mainly due to extensive lateral root development (Figure 3A).

### Overexpression of *CmF-308* in the phloem alters root and shoot development of tomato plants

**Figure 4:**
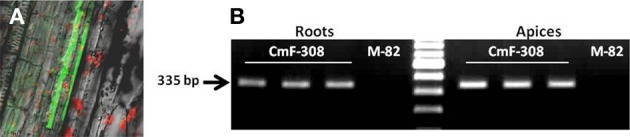
**(A)** Activity of *AtSUC2* promoter in transgenic tomato plants. GFP is visualized the vasculature (companion cells) of tomato stem. **(B)** Presence of *CmF-308* transcript in shoot apices and roots of three independent transgenic tomato plants expressing the gene under the *AtSUC2* promoter. The commercial variety M-82 served as a control for the RT-PCR assay.

**Figure 5:**
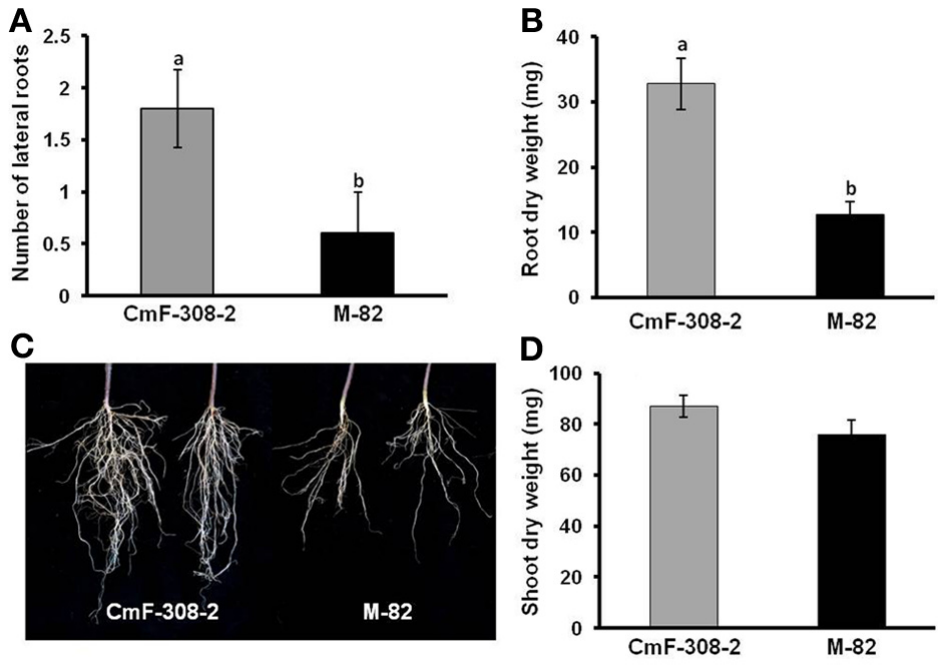
**Effect of *CmF-308* expression in the phloem of tomato plants on root development.** Number of lateral roots **(A)**, root dry weight **(B)**, and a picture **(C)** presenting the differences between roots of transgenic tomato plants expressing *CmF-308* under the *AtSUC2* promoter (CmF-308-2) as compared with the control variety M-82. **(D)** Shoot dry weight of transgenic tomato plants expressing *CmF-308* under the *AtSUC2* promoter (CmF-308-2) as compared with the control variety M-82. Lateral roots were counted in 9-days old seedlings germination on germination papers **(A)**. Shoot and root weight was measured in 3-week old pot-grown plants **(B–D)**. Data represent means of six replications ± SE. Different letters indicate significant differences between the plant lines at *p* < 0.05 by Student's *t*-test.

To further explore the effect of phloem-limited expression of *CmF-308* on plant development, the gene's coding sequence was inserted into transgenic tomato plants under the control of the phloem specific *AtSUC2* promoter (Shalit et al., 2009). Activity of the *AtSUC2* promoter, as phloem specific promoter in tomatoes was verified using GFP as a reporter protein (Figure 4A). In agreement with the activity of *AtSUC2* promoter along the vasculature, *CmF-308* was expressed in the shoot apices and roots of the transgenic tomato plants (Figure 4B). Over ten independent homozygous *CmF-308*-transgenic tomato plants were generated. Most of them had similar phenotype and two representative lines were selected for further study. Similar to the effect observed after transient expression, constitutive expression of *CmF-308* in the phloem of tomato plants caused a significant increase in the number of lateral roots and root weight (Figures 5A–C). Note that shoot weight was similar in *CmF-308* and control tomato plants (Figure 5D).

**Figure 6:**
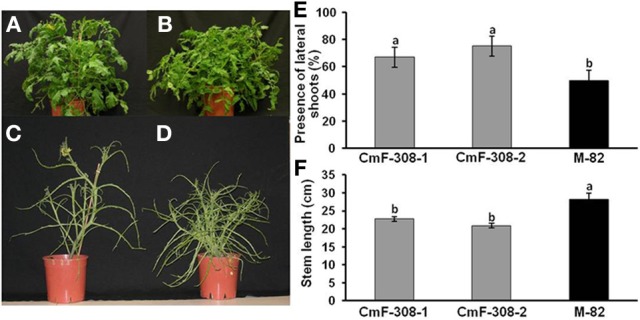
**Effect of *CmF-308* expression in the phloem of tomato plants on shoot development.** Control variety M-82 **(A,C)** as compared with transgenic plants expressing *CmF-308* under the *AtSUC2* promoter **(B,D)**. Pictures were taken 60 days after germination. Plants were stripped of all their leaflets **(C,D)** to assist in visualizing the branching phenotype. Percentage of lateral shoots **(E)** and stem length **(F)** of the control tomato variety M-82 and two transgenic lines expressing *CmF-308* under the *AtSUC2* promoter, 30 days after germination. Data represent means of 12 replications ± SE. Different letters indicate significant differences between the plant lines at *p* < 0.05 by Student's *t*-test.

In addition to the influence on root development, phloem-specific expression of *CmF-308* significantly affected shoot development (Figure 6). Transgenic plants expressing *CmF-308* in the phloem were significantly shorter (Figure 6F) and had a higher number of axillary shoots than the control tomato variety. Whereas in the control variety, lateral shoots developed in about 50% of the nodes, the percent of lateral shoots in the transgenic plants was between 70 and 80% (Figure 6E). The axillary shoots were longer, exhibiting a typical phenotype of reduced apical dominance (Figures 6A–D). In addition, these transgenic plants were characterized by delayed flowering.

### Overexpression of *CmF-308* in the phloem affects auxin sensitivity

**Figure 7:**
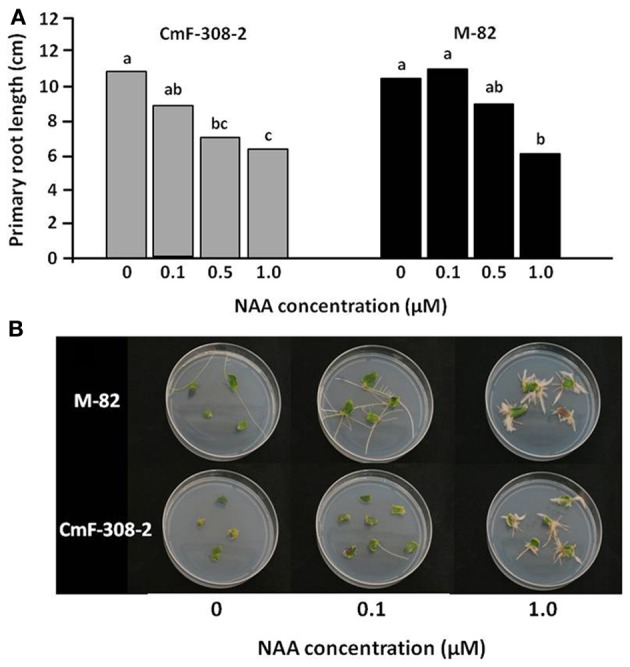
**Auxin response of *CmF-308*-transgenic tomato plants. (A)** Primary root length of CmF-308-2 and control M-82 tomato seedlings after incubation in various concentrations of NAA. Seedlings were germinated on filter paper soaked in the auxin solutions in the dark. Lengths of primary roots were measured 10 days after germination. Data represent means of eight replications ± SE. Different letters indicate significant differences between auxin concentration treatments in each plant line Axillary at *p* < 0.05 by Tukey's HSD-test. **(B)** Auxin dose-response assay of cotyledon explants showing reduced auxin sensitivity in CmF308-2 cotyledons as compared to controls. Root regeneration is promoted by 0.1 μM NAA in the control variety and by 10 times higher concentration (1.0 μM NAA) in the transgenic line CmF-308-2. Pictures of representative plates were taken 12 days after placing the cut cotyledons on the various auxin media.

The changes in lateral root and axillary shoot development suggested altered sensitivity to auxin in plants expressing *CmF-308* under a phloem-specific promoter. We therefore examined the sensitivity of roots and shoots of transgenic and control plants to auxin. A dose-response assay revealed significant inhibition of primary root lengthening in *CmF-308* plants at a concentration of 0.5 μM NAA, with no significant effect of this concentration on root length of control tomato plants (Figure 7A). This indicated that the roots of plants expressing the *CmF-308* gene product are more sensitive to exogenous auxin than control roots. Primary root elongation of both control and CmF-308 plants was significantly inhibited by 1 μM NAA.

Additional assay was aimed to examine the auxin response in shoot tissue. Cotyledon segments were subjected to various NAA concentrations and the number of developing roots was monitored. Interestingly, substantial rooting was evident in control cotyledons subjected to 0.1 μM NAA, whereas only a negligible number of roots developed from cotyledons of *CmF-308*-transgenic plants (Figure 7B). Similar differences in rooting level could be observed when the cotyledons were subjected to 1 μM NAA.

Collectively, these results indicated that overexpression of *CmF-308* in the phloem of tomato plants causes a decrease in the shoot segments' sensitivity to auxin and an increase in roots' sensitivity.

### Overexpression of *CmF-308* in the phloem affects hormone-related gene expression

**Figure 8:**
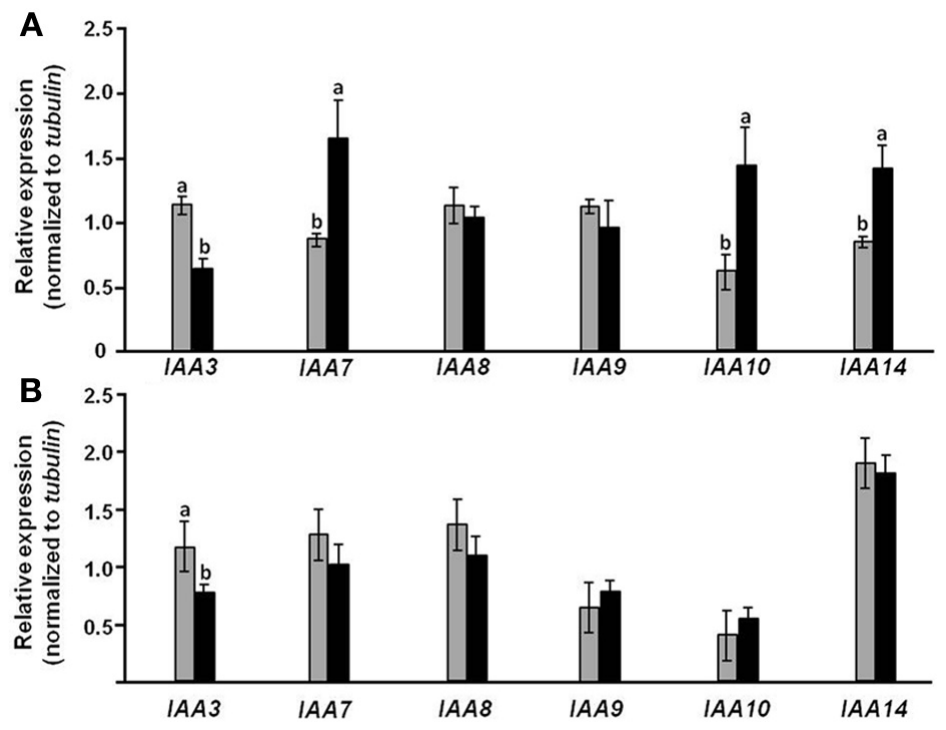
**Effect of *CmF-308* expression in the phloem of tomato plants on the expression of auxin-response genes.** Expression of members of the *Aux/IAA* gene family was determined by qRT-PCR in leaves **(A)** and roots **(B)** of 21-day-old *CmF-308*-transgenic (gray columns) and control M-82 (black columns) plants. Data represent means of four replications ± SE. Different letters indicate significant differences in relative expression of specific genes between the two plant lines at *p* < 0.05 by Student's *t*-test.

It is logical to assume that tissue-specific alteration in auxin sensitivity due to overexpression of *CmF-308* in the phloem is associated with changes in related genes' expression. To further explore the mode by which *CmF-308* expression affects auxin sensitivity, relative expression of various *Aux/IAA* transcripts was analyzed in leaves and roots of *CmF-308*-transgenic and control tomato plants. Consistent with reduced sensitivity to auxin, relative expression of the tomato *IAA7*, *IAA10* and *IAA14* was significantly lower in the leaves of *CmF-308*-transgenic vs. control plants (Figure 8A). Interestingly, relative expression of *IAA3* was higher in *CmF-308* vs. control leaves, while no significant differences were observed in the relative expression of *IAA8* and *IAA9* between leaves of the two tomato lines.

Relative expression of most examined *Aux/IAA* transcripts was similar in the roots of transgenic and control tomato plants. An exception was *IAA3*, whose relative expression was significantly higher in roots of *CmF-308* vs. control plants (Figure 8B).

**Figure 9:**
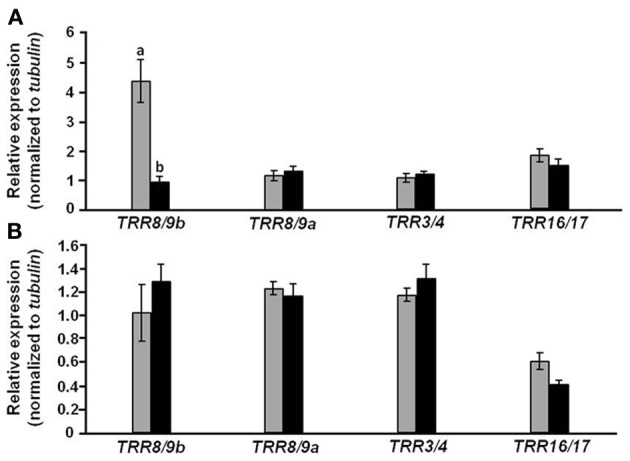
**Effect of *CmF-308* expression in the phloem of tomato plants on the expression of cytokinin-response genes.** The expression of members of the tomato response regulator (*TRR*) gene family was determined by qRT-PCR in leaves **(A)** and roots **(B)** of 21-day-old *CmF-308*-transgenic (gray columns) and control M-82 (black columns) plants. Data represent means of four replications ± SE. Different letters indicate significant differences in relative expression of specific genes between the two plant lines at *p* < 0.05 by Student's *t*-test.

Further study was aimed at verifying the interaction between expression of auxin- and cytokinin-responsive genes (Figure 9). Relative expression of four cytokinin-induced *Type A-tomato response regulator*s (*TRR*s) was similar in roots of *CmF-308*-transgenic and control plants (Figure 9B). However, relative expression of *TRR8/9b* was almost five times higher in leaves of *CmF-308* vs. control plants (Figure 9A), suggesting a higher cytokinin response associated with the observed reduced auxin response.

One of the most pronounced characteristics of *CmF-308*-transgenic plants was massive branching, indicating reduced apical dominance. A major regulator of axillary shoot development is the branching inhibitor strigolactone (Gomez-Roldan et al., 2008). It is generally assumed that strigolactone is synthesized in the roots and move via the xylem into the shoots. We therefore, examined whether expression of genes coding for strigolactone biosynthesis is altered in the roots of *CmF-308*-transgenic plants. Relative expression of *CCD7* (*carotenoid cleavage dioxygenase*), a gene coding for one of the key enzymes involved in strigolactone biosynthesis, was significantly (about twofold) higher in the roots of *CmF-308* plants vs. controls (Data not shown). Relative expression of *CCD8* was similar in roots of *CmF-308* and control plants. These results suggested that enhanced branching in *CmF-308*-transgenic plants is not associated with inhibition of strigolactone biosynthesis.

## Discussion

A previous study established that two melon *Aux/IAA* transcripts are capable of long-distance movement from melon rootstock to pumpkin scion (Omid et al., 2007). The ability of one melon *Aux/IAA* transcript, *CmF-308*, to move long distances was further established here in *N. benthamiana* plants (Figure 2). The presence of *CmF-308* in the shoot apex 48 h after agroinfiltration indicated that this transcript has the ability to move from cell to cell and enter the companion cell–sieve element (CC–SE) complex. It is logical to assume that such trafficking requires a chaperoning mechanism, probably as a ribonucleoprotein (RNP) complex. This notion is supported by the fact that phloem sap contains numerous RNA-binding proteins (Giavalisco et al., 2006; Lin et al., 2009), and by the demonstrated *in vitro* interaction between phloem sap-specific proteins and RNA molecules (Yoo et al., 2004; Ham et al., 2009). The absence of *gfp* transcripts from tissues outside the infiltrated leaf indicates that cell-to-cell and long-distance movement of mRNA are characteristic of specific molecules. Higher accumulation of *CmF-308* in the veins and vascular bundles (Figure 1) suggests that this transcript is indeed destined for long-distance movement. The biological role for the long-distance trafficking of *Aux/IAA* transcript has yet to be explored.

To further explore the significance of *CmF-308* expression in the vascular tissue, the gene was expressed in transgenic tomato plants under the *AtSUC2* promoter. Various mutations in *Arabidopsis Aux/IAA* genes result in minor or no phenotypic changes, suggesting functional redundancy among Aux/IAA members (Overvoorde et al., 2005). However, antisense silencing of *SlIAA9* (Wang et al., 2005) or *SlIAA3* (Chaabouni et al., 2009) affected leaf architecture, root and fruit development. One should remember that the above-described *Aux/IAA* antisense constructs were inserted into tomato plants under the *CaMV-35S* promoter. The substantial phenotypic changes in tomato plants expressing *CmF-308* predominantly in the phloem indicate that developmental processes are affected by altered auxin response imposed by the CC–SE complex. In this respect, it is important to note that we made numerous attempts to insert an antisense construct of *CmF-308* into tomato plants under the *AtSUC2* promoter. None of these attempts enabled regeneration of even one transgenic tomato plant, suggesting that silencing *Aux/IAA* in the phloem might be lethal.

Interestingly, overexpression of *CmF-308* under a phloem-specific promoter resulted in significant modification of both root and shoot development (Figures 5, 6). Root development was altered in Arabidopsis and tomato plants in which various *Aux/IAA* genes were either mutated or silenced. For example, dominant Arabidopsis mutants of *IAA19* (Tatematsu et al., 2004) or *IAA14* (Fukaki et al., 2002) were characterized by few or complete lack of lateral roots, respectively. Similarly, gain of function mutations in *IAA28* and *IAA18* also resulted in defected formation of lateral root (Rogg et al., 2001; Uehara et al., 2008). However, a dominant *IAA7* Arabidopsis mutant, had more lateral roots then control plants (Nagpal et al., 2000). These results indicate that different *Aux/IAA* gene products have contrasting effect on root growth and lateral root formation. A comparison of *CmF-308* coding sequence and its Arabidopsis and tomato homologs revealed that the closest Arabidopsis homologs are *IAA14* (1e-98) *IAA7* (2e-89), *IAA16* (4e-80) and *IAA17* (6e-78) while the closest tomato homologs are *IAA14* (5e-128) *IAA9* (2e-108) *IAA16* (7e -108) and *IAA7* (3e-100). Due to the high degree of similarity between the indicated *Aux/IAA* genes, one cannot determine which one is the *CmF-308* ortholog. It is possible that functioning of the *CmF-308* gene product resembles the functioning of *AtIAA7*, namely enhanced formation of lateral roots. In this respect it is important to note that silencing *SlIAA9* in transgenic tomato plants, expressing the antisense construct, enhanced auxin sensitivity and resulted in higher number of lateral roots (Wang et al., 2005). Interestingly, this phenotype was associated with upregulation of *SlIAA3* expression predominantly in the vasculature (Chaabouni et al., 2009). Expression of *SlIAA3* was upregulated in roots of our *CmF-308* plants, raising the possibility that overexpression of *Cm-F308* in the phloem exerts an effect on lateral root formation via upregulation of *SlIAA3*.

Auxin-response assays confirmed that auxin sensitivity of *CmF-308* plants is altered in a tissue-specific manner (Figure 7): roots of *CmF-308* plants were indeed more sensitive to auxin than control roots, but auxin sensitivity of *CmF-308* shoot tissue (cotyledons) was lower than that of control plants. These findings suggest that *CmF-308* can both repress and activate auxin responses in tomato plants.

Auxin responses in *Arabidopsis* plants expressing either gain-of-function or loss-of-function mutations in *IAA3* indicated that this gene's product acts as both a positive and negative regulator of the auxin response (Tian and Reed, 1999). Those authors suggested that weak transient auxin signaling induces a low level of *IAA3* in the roots which is sufficient to promote root growth and lateral root formation. However, stronger auxin signaling induces a higher level of *IAA3*, inhibiting these responses. This dual function of *Aux/IAA* is supported by previous studies demonstrating that the auxin response is dose-dependent, with stimulation of root growth by low concentrations of exogenous auxin and inhibition of root growth by higher IAA concentrations (Evans et al., 1994). Similarly, one can suggest that the *CmF-308* gene product accumulates at higher levels in the shoot apex, resulting in reduced auxin response, inhibition of apical dominance and enhanced development of lateral shoots. In contrast, low levels of *CmF-308* in the root cause a slight increase in *SlIAA3*, thereby enhancing the auxin response and lateral root formation. The finding that light regulates *IAA3* expression (Tian et al., 2002) suggests a lower expression level of this transcript in root compared to shoot tissues, resulting in a differential auxin response.

An additional explanation for the effect of *CmF-308* on plant development might relate to the expression of other genes mediating responses to growth substances. The expression of three *Aux/IAA* genes (*IAA7*, *IAA1*0 and *IAA14*) was lower in leaves of *CmF-308* vs. control plants, in line with the lower auxin response in the former. It is important to note that in parallel to reduced expression of these three *Aux/IAA* genes, expression level of *TRR8/9b* was significantly higher in *CmF-308* leaves (Figure 8). Expression of *TRR8/9b* was upregulated by cytokinin (Shani et al., 2010), suggesting that the effect of the *CmF*-*308* gene product on apical dominance and shoot branching is via modulation of the cytokinin response. Reduced expression of *IAA7* was recently found in *Arabidopsis* plants paralleling an increase in the cytokinin response (Brenner and Schmülling, 2012); this supports the notion of cross talk between auxin- and cytokinin-signaling pathways.

The presented results provide support for *Aux/IAA* functioning in the phloem. The altered developmental program of cells distant from the CC–SE complex suggests an involvement of a phloem borne signal mediating auxin response. Future study should aim to identify potential interacting proteins (molecules) that might be associated with the short- or long-distance trafficking of the *Aux/IAA* gene product.

### Conflict of interest statement

The authors declare that the research was conducted in the absence of any commercial or financial relationships that could be construed as a potential conflict of interest.

## Appendix

**Table A1:** **List of primers used for amplification of the examined genes**.

**Amplified gene**	**Forward primer (5′ → 3′)**	**Reverse primer (5′ → 3′)**
*CmF-308-3XHA*	GGG CCAAG AATGATAG AC	CTA CTG AGCAGCGTA ATCTGG
*TRR8/9b*	AGTATGCCGGAAATGACTGG	TGGAACATTTTCCGATGACA
*TRR16/17*	GGTCTAAGGGCGTTGGAGTA	TCCTGGCATGCAATAATCTG
*CCD7*	TGGGAAGGTGGTGATCCTTA	TAGCTGAGCAGCAACATCCA
*CCD8*	CAATCACAGCGGTAACTCTTCCA	GCATCCTGATTCTAAAGCATTT
*CmF-308*	GACTGGAGTTACCGTCGATCT	CGGAGTCAGGGCTCTTTTGA
*SlIAA14*	CCTGAAGTTCATCTGCACCA	GTTCACCTTGATGCCGTTCT
*SlIAA9*	CAAATACGTGAAGGTAGCAGTTGAC	ACACCATTTGTAAGGTCCATAAGCT
*SlIAA3*	GACTTCTCAAAAGCTTGATCGAGAG	TGAAATCTTTCATTCCTTGGACAA
*SlIAA7*	AGCCACCAACTAAGGCTCAA	CCATCCATGGAAACCTTCAC
*SlIAA8*	CAAATACGTGAAGGTAGCAGTTGAC	ACACCATTTGTAAGGTCCATAAGCT
*SlIAA10*	GACTTCTCAAAAGCTTGATCGAGAG	TGAAATCTTTCATTCCTTGGACAA
*TRR3/4*	CGTCCCCTAAAGCATTCTCA	CGTCTTGTTGGTGATGTTGG
*TRR8/9a*	TGCTTAGAAGAAGGGGCAGA	GGGGGCTTTTACATTTGGTT
*SlTubulin*	GAAAGCCTACCATGAGCAGC	CTTTGGCACAACATCACCAC
